# Applied insight: studying reducing the carbon footprint of the drying process and its environmental impact and financial return

**DOI:** 10.3389/fbioe.2024.1355133

**Published:** 2024-03-14

**Authors:** Ayman Ibrahim, Alia Amer, Islam Elsebaee, Amr Sabahe, Mariam A. Amer

**Affiliations:** ^1^ Bioengineering Department, Agricultural Engineering Research Institute (AEnRI), Agricultural Research Center (ARC), Giza, Egypt; ^2^ Medicinal and Aromatic Plants Research Department, Horticulture Research Institute, Agricultural Research Center (ARC), Giza, Egypt

**Keywords:** drying, solar energy, hybrid solar dryer, energy consumption, greenhouse gas emissions, carbon footprint

## Abstract

Harnessing solar energy is one of the most important practical insights highlighted to mitigate the severe climate change (CC) phenomenon. Therefore, this study aims to focus on the use of hybrid solar dryers (HSDs) within an environmentally friendly framework, which is one of the promising applications of solar thermal technology to replace traditional thermal technology that contributes to increasing the severity of the CC phenomenon. The HSD, based on a traditional electrical energy source (HS_TEE_) and electrical energy from photovoltaic panels (HS_PVSE_), was evaluated compared to a traditional electrical (TE) dryer for drying some medicinal and aromatic plants (MAPs). This is done by evaluating some of the drying outputs, energy consumed, carbon footprint, and financial return at 30, 40, and 50°C. The best quality of dried MAP samples in terms of essential oil (EO, %) and microbial load was achieved at 40°C. The HS_TEE_ dryer has reduced energy consumption compared to the TE dryer by a percentage ranging from 37% to 54%. The highest CO_2_ mitigated ratio using the HS_TEE_ dryer was recorded in lavender, thyme, basil, lemongrass, and sage samples with values ranging from 45% to 54% at 30, and 50°C. The highest financial return obtained from energy consumption reduction and carbon credit footprint was achieved at 50°C, with values ranging from 5,313.69 to 6,763.03 EGP/year (EGP ≈ 0.0352 USD) when coal was used as a fuel source for the generation of electricity. Moreover, the HS_PVSE_ dryer achieved a 100% reduction in traditional energy consumption and then reduced CO_2_ emissions by 100%, which led to a 100% financial return from both energy reduction and carbon credit. The highest financial returns were observed at 50°C, with values ranging from 13,872.56 to 15,007.02, 12,927.28 to 13,984.43, and 11,981.99 to 12,961.85 EGP/year (EGP ≈ 0.0352 USD) for coal, oil, and natural gas, respectively. The HS dryers show potential for environmental conservation contribution; furthermore, earning money from energy savings and carbon credit could help improve the living standards and maximize benefits for stakeholders.

## 1 Introduction

Traditional, non-renewable energy sources that rely on different types of fuels such as coal, oil, and natural gas for generation and their effective contribution to the risks of climate change (CC) that threaten the environment and achieving sustainable development have become a global concern. Warming is a global phenomenon that has caused very serious environmental problems in the world in the last few decades. Various types of greenhouse gases (GHGs) exist, and their global warming potential varies. Carbon dioxide (CO_2_), nitrous oxide (N_2_O), methane (CH_4_), carbon monoxide (CO), and others are among the most important GHGs emitted into the atmosphere that lead to heating Earth. CO_2_, CH_4_, and N_2_O are naturally occurring gases in the atmosphere and are also generated through different human activities (Garg et al., 2006; McMichael et al., 2006; Hann et al., 2020; Preble et al., 2020). According to the European Parliament (2021), CO_2_ represented almost 80% of the volume of all GHG emissions that contribute to global warming phenomena, followed by methane (CH_4_) with more than 12% (Gavurova et al., 2021). The massive steady increase in population pushed the world to double agricultural production and food processing to meet this huge population increase. As a result, there has been a huge increase in the demand for energy, which, in turn, has led to an increase in CO_2_ emissions, thus increasing the phenomenon of severe climate change. The Climate Change Conference COP27 and COP28 in Egypt and the United Arab Emirates, respectively, showed their commitment to combating climate change by updating its plan toward nationally determined contributions (NDCs). The Egyptian government has enhanced ambitions toward increasing its main goal of reducing GHG emissions generated from electricity generation, transmission, and distribution from 33% to 37% reduction by 2030. Furthermore, promised to forward its target date of generating 42% of its energy from renewable energy by 2030 instead of 2035 (European Parliament, 2021). Generally, the United States Energy Information Administration (EIA, 2014**)** mentioned that the industrial sector devours a major portion of the energy produced to meet consumer needs. It consumes the largest share of energy, reaching 54% of the total energy in the world. Furthermore, it categorized the industries according to their energy needs as energy-intensive, non-energy-intensive, and non-industrial manufacturing. The Food and Agriculture Organization estimated that approximately 30% of the world’s demand for energy is used by the food sector. Most of this energy is used for the needs of energy-intensive post-harvest treatments and food processing, such as washing, drying, cooling, storage, and extraction (FAO, 2020). The food sector consumes approximately 200 exajoules (EJ) annually, of which 45% is related to food processing and distribution. This huge amount of energy consumed by the food industry surely is associated with high levels of GHG emissions and natural resource depletion (FAO, 2020; EIA, 2020; Sims et al., 2020; FAO, 2021). The majority of the total energy consumed (80.2%) is generated by fossil fuels **(IEA, 2019)**. The food industry still relies on various forms of fossil energy sources, and therefore, the current industries used in food production are unsustainable and do not achieve food security in addition to creating serious environmental risks **(** Kucukvar and Samadi, 2015; Holden et al., 2018; Bamisile et al., 2020; Keller et al., 2018; Mathur et al., 2022; Saleem, 2022 **)**. As a result, Ritchie and Roser (2021) pointed out that the CO_2_ emitted each year is estimated to be 36 billion tons, which is a massive contribution to environmental pollution. Meanwhile, Odell (2000) mentioned that it is predicted that fossil fuels may be depleted by 2060. Therefore, the replacement of fossil fuels with renewable energy is a necessary priority. Drying agricultural products is one of the most important food processes aimed at preserving food and extending its shelf life to achieve sustainability and food security (Kumar et al., 2014; Calín-Sánchez et al., 2020; Matavel et al., 2022; Palumbo et al., 2022; Petikirige et al., 2022; Ibrahim et al., 2023a). Traditional drying methods that operate based on traditional energy sources, whether electric energy or fossil fuels, consume excessive energy and, thus, emit huge amounts of GHGs (Motevali et al., 2014; Sivakumar et al., 2016; Motevali and Koloor, 2017; Kaveh et al., 2020; Kaveh et al., 2021; Matavel et al., 2022; Ibrahim et al., 2023a). The most important disadvantages of traditional dryers is not only the massive depletion of non-renewable energy sources but also their low efficiency as continuous exposure of products to high temperatures for long periods leads to deterioration of quality characteristics (Kumar et al., 2005; Sreekumar et al., 2008; Bi et al., 2015; Maisnam et al., 2017; Lingayat et al., 2020; Udomkun et al., 2020; Vanlauwe and Müller, 2020; Ibrahim et al., 2023a). Therefore, one of the most important solutions to reducing GHG emissions in the food processing sector is to replace these traditional drying systems that deplete traditional energy sources with modern and innovative systems that rely on accurate control systems and use renewable energy sources, where renewable energy sources aim to reduce the traditional energy consumed or rely on clean energy only, which, in turn, leads to a great reduction in GHG emissions. On the other hand, modern drying methods such as microwave, vacuum, infrared, and freeze drying lead to higher quality but consume a lot of energy. Although traditional open-sun drying does not consume any energy, it has many disadvantages such as food loss and a sharp decrease in quality (Vadivambal and Jayas et al., 2010; Dak and Pareek, 2014; Zielinska et al., 2015; Belwal et al., 2022; Wardhani et al., 2022; Ibrahim et al., 2023a). Solar drying systems, whether they depend solely on solar energy or are self-controlled hybrid drying systems, can achieve a significant reduction in CO_2_ emissions as a result of a considerable reduction in the traditional energy consumed. In this regard, Ibrahim et al. (2023b) developed a hybrid smart solar dryer (HSSD) based on indirect forced air convection via solar collectors and a controlled auxiliary heating system. The results achieved an increase in the drying rate, remarkably saving energy from 25.54% to 77.1% vs. the traditional drying technique, and providing high-quality products. Solar thermal systems are widely applied in the domestic sector and also provide huge potential and benefits for industries. Hansen and Vad Mathiesen (2018) mentioned that the solar thermal role is to lower the burden on scarce renewable resources and to supply renewable energy under conditions where no alternatives are available. Gudiño-Ayala and Calderón-Topete (2014) concluded that the solar mode dryer requires, on average, 31.2% longer drying time than the dryer operated in the hybrid mode under similar conditions. A life-cycle assessment of industrial solar thermal systems in Europe was conducted by Kylili et al. (2018), who found considerable savings in both energy and CO_2_, which range from 35 to 75 GJ and 2 to 5 tons of CO_2_/kWth. Ndukwu et al. (2021) studied the solar drying of medicinal and aromatic plants, and the results showed that there is a reduction in CO_2_ emissions from 40,702.38 to 407,023.8 kg/year in the drying of grapes in Egypt and 2,308.5 to 23,085 kgCO_2_/year for drying of chili in Nigeria. Moreover, the hybrid dryers had a greater quality than open-air sun-drying, and it had diminished 31.8 tons of CO_2_. Furthermore, many investigations concluded that hybrid dryers used on a variety of agro-products were ideal for drying crops quickly while maintaining great product quality (Ferreira et al., 2007; Eltawil et al., 2018; Capossio et al., 2022; Jahromi et al., 2022; Ibrahim et al., 2023a; Suresh et al., 2023). Undoubtedly, the industrial sector devours the largest share of the energy produced and, thus, contributes a large percentage to the increase in GHGs. The food processing sector, which contains various and multiple processors for all agricultural products to produce food to meet and secure the nutritional needs of the greatly increasing population, contributes the largest share in the increase in GHGs. Drying agricultural products is one of the most important processes of these food processors. Certainly, the traditional drying methods that operate based on traditional energy sources, whether electric energy or fossil fuels, emit huge amounts of GHGs. Therefore, this study aims to rely on renewable energy (solar energy) in the solar hybrid mode in a controlled way that aims to reduce the total energy consumption and the carbon footprint of the drying process. This will be achieved through estimating energy consumption and CO_2_ emissions compared to traditional drying techniques. Hence, the achievement of this study helps fulfill Egypt’s commitment to combating CC through its updated plan toward NDCs, which focuses on forwarding its target date of generating 42% of its energy from renewable energy by 2030 instead of 2035 and increasing the reduction in GHG emissions generated from electricity generation, transmission, and distribution from 33% to 37% by 2030.

## 2 Materials and methods

This methodology will focus on the analysis of the energy consumed to carry out the drying process. Hence, to estimate the amount of CO_2_ generated, a hybrid solar drying system based on traditional electrical energy (HS_TEE_), a hybrid solar drying system based on photovoltaic panels that use solar energy as a source of electrical energy (HS_PVSE_), and a traditional electric dryer (TE) are three different drying systems that were used to conduct this methodology.

### 2.1 Case study: smart drying using different drying systems

**FIGURE 1:**
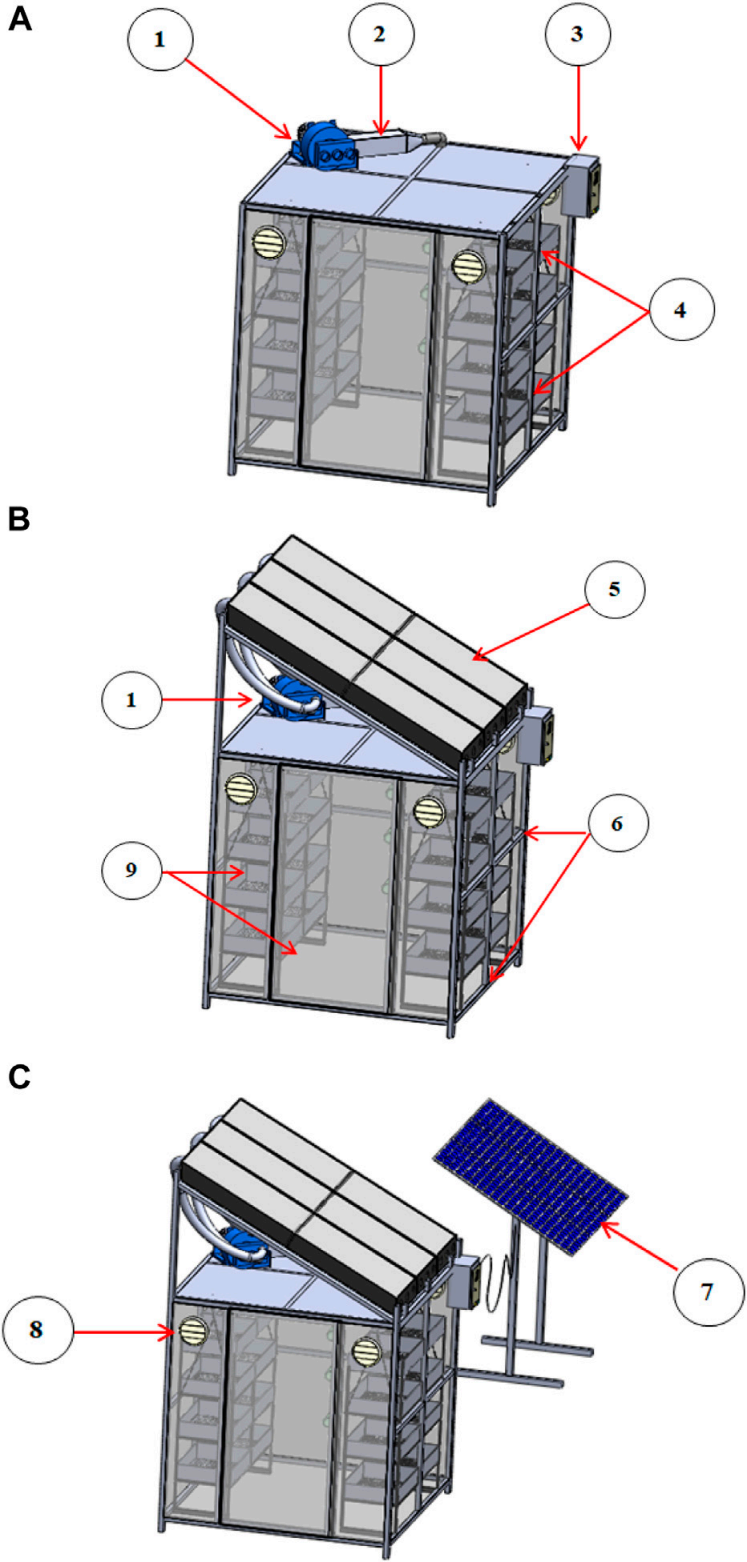
Isometry of the dryer with three different energy supply sources: **(A)** traditional electric dryer (TE dryer), **(B)** hybrid solar dryer that uses traditional electric energy (HS_TEE_ dryer), **(C)** hybrid solar dryer based on the proposed photovoltaic solar energy (HS_PVSE_ dryer). (1) Blower, (2) heater chamber, (3) control unit, (4) drying trays, (5) solar collector, (6) drying chamber chassis, (7) solar panels, (8) suction fan, and (9) polycarbonate sheet.

This case study was carried out on parts of lemongrass (*Cymbopogon citratus*), thyme (*Thymus vulgaris*), marjoram (*Origanum majorana*), lavender (*Lavandula dentata*), sage (*Salvia Officinalis* L.), and basil (*Ocimum basilicum* L.). These fresh herbs were obtained from the farm of the Medicinal and Aromatic Plants Research Department, Horticulture Research Institute, Agricultural Research Center (ARC), Egypt, in August 2022. This smart-drying case study consists of using a smart dryer that operates with three different operating systems, as shown in Figure 1. The first system is the TE dryer, which consists of a drying chamber, a heating unit (electric heaters), blower, and a control unit to monitor the temperature and relative humidity (Figure 1A); details of design parameters, specifications, and fabrication stages were described by Ibrahim et al. (2023b). The second drying system is a hybrid solar dryer that uses traditional electricity as the energy source (HS_TEE_). This HS_TEE_ is composed of the TE dryer attached to the solar collector, as shown in Figure 1B. The third drying system is based on the proposed photovoltaic panels as a source of generating electrical energy (HS_PVSE_), which consists of the TE and HS_TEE_ dryers attached to the photovoltaic panel unit to generate the electrical energy, as shown in Figure 1C. All experiments were conducted in the climatic location of the Al-Qanater Al-Khairiya area, Qalyubia Governorate, Egypt, 30°19′N, 31°13′E, 16.9 m above sea level, and the average solar radiation, air temperature, humidity, and wind speed were taken into account. These climatic parameters were measured and recorded every 30 min during the day. A solar radiation station (model: RK200-05, spectral range 300–1100 nm) was used for measuring solar radiation (W/m^2^). Furthermore, relative humidity (RH), temperature, and wind speed were measured using the environment meter multi-function (model: EM-9300SD) with a humidity/temperature range of 0%–95% RH and a temperature range of 0°C–50°C, with an accuracy of 70% RH: ≧ ± (3% reading + 1% RH), (< 70% RH: ± 3% RH), ± 0.8°C. The wind speed was measured using an anemometer ranging from 0.4 to 25.0 m/s, with accuracy 2% + 0.2 m/s. Finally, to obtain the electric current required to operate the different drying systems, a hand-held 3 5/6-bit digital multimeter (UNI-T UT89X) was used to measure the voltage, AC voltage, 10,000 V; DC voltage, 1,000 V AC; and current, 20 A DC current; then, the power requirement was calculated (Ibrahim et al., 2023b **)**. The idea of operating the dryer depends mainly on the control unit to monitor the drying process sequence steps to start exploiting solar radiation to increase the air temperature within the solar collector until the temperature inside the solar collector reaches 60°C and then force it into the drying chamber till the required drying temperature is achieved, i.e., 30 or 40 or 50°C, and then stops.

In the case of a decrease in the solar radiation intensity to increase the air temperature inside the solar collectors and then increasing the air temperature inside the drying chamber to the required drying temperature, the air from the solar collectors is forced onto the auxiliary heating system consisting of the electric heater until the required drying temperature is achieved.

### 2.2 Energy consumption analysis

All the power requirements needed to operate electric heaters, blowers, and air fans were calculated according to Eq. 1:

P=I×V (1)

where P is the output electrical power (watts, W), V is the voltage in volts, and I is the electric current in amperes. Subsequently, the time required (h) to operate each device that needs electrical power is measured. Energy consumption for the dryers used in this investigation was analyzed according to three different types of dryers: TE dryer, HS_TEE_ dryer, and HS_PVSE_ dryer. The energy consumptions were calculated according to the following sequence equations (Ibrahim et al., 2023b **)**:

$$E.C.S.S.=\left(BP\times t_{B}\right)+\left(PFP\times t_{FP}\right)+\left(SFP\times t_{SF}\right)+\left(FFP\times t_{FF}\right)$$ (2)

where E.C.S.S. is the energy consumed by the solar system (kWh); BP is the blower power (kW); $t_{B}$ is the time (min) for operating the blower; and PFP , SFP , and FFP and $t_{FP}$ , $t_{SF}$ , and $t_{FF}$ are the power (kW) and time (min) required for push, suction, and flipping air fans, respectively. If the solar collector cannot supply the drying chamber with the necessary amount of heat, the auxiliary heating system is operated with the heating system using the solar collector, and the energy consumption is calculated according to Eq. 3, where this energy consumption for the TE dryer and HS_TEE_ dryer is

$$E.C.S.AH.S.=E.C.S.S.+\left(HP\times t_{AH}\right)$$ (3)

where E.C.S.AH.S. is the energy consumed by the solar system and the auxiliary heating system (kWh) (hybrid solar dryer); HP is the heater power (kW); and $t_{AH}$ is the time required for operating the auxiliary heating system.

### 2.3 CO_2_ emission analysis

The amounts of CO_2_ emissions emitted as a result of the operation of the dryer were estimated based on the amount of energy consumed to operate dryers with different energy sources. The amounts of CO_2_ emissions were calculated according to the classification of dryers based on the different energy sources:

• Traditional electric dryer (TE dryer),
• Hybrid solar dryer uses traditional electric energy (HS_TEE_ dryer),
• Hybrid solar dryer based on the proposed photovoltaic solar energy (HS_PVSE_ dryer) as the energy source.

Below is a sequence of applied equations to calculate the amounts of CO_2_ emissions resulting from the operation of the TE, HS_TEE_, and HS_PVSE_ dryers. The daily energy consumed by the TE, HS_TEE_, and HS_PVSE_ dryers was determined in terms of the power required and the operation time (Elhage et al., 2018) shown as follows in Eq. 4:

$$E_{cd}=P_{r}\times T_{dt}$$ (4)

where $E_{cd}$ is the energy consumed per day (kWh/day) of the product, $P_{r}$ is the power required (kW), and $T_{dt}$ is the total drying time per day of the product (hr/day). Using the results obtained from Eq. 4, it is possible to calculate the energy consumed to dry the products under study throughout the year by assuming that the number of working days per month is 20 and that it will be operated over 12 months per year. Therefore, using Eq. 5, the energy consumed throughout the year is estimated.

$$E_{{c/}_{year}}=E_{cd}\times W_{days/month}\times 12$$ (5)

where $E_{{c/}_{year}}$ is the energy consumed for drying/year (kWh/year) and $W_{days/month}$ is the working period/month. Based on the estimates of the traditional electrical energy consumed by dryers throughout the year to complete drying operations and link it to the different types of fuel (coal, oil, and natural gas) consumption used to generate this electrical energy, the amount of CO_2_ emitted to generate this electrical energy can be calculated and estimated through the following equation:

$$M_{CO2}=E_{{c/}_{year}}\times {EF}_{{CO}_{2}}$$ (6)

where $M_{CO2}$ is the mass of CO_2_ produced by different types of fuel consumption and ${EF}_{{CO}_{2}}$ is the CO_2_ emission factor (kgCO_2_/kWh). ${EF}_{{CO}_{2}}$ was estimated according to the CO_2_ amount released from fuel combustion to generate kWh of electrical energy. As there are various sources of fuel for producing electrical energy in Egypt, ${EF}_{{CO}_{2}}$ for each type is listed in Table 1, according to the International Energy Agency (IEA, 2016). Subsequently, the amount of CO_2_ that was mitigated for hybrid solar drying and the reduction percentage compared to the conventional drying system based on electrical energy were calculated using Eqs 7, 8:

$$M_{CO2\;Reduced}=M_{CO2\;Electric}-M_{CO2\;Hybrid\;}$$ (7)

$$M_{{CO2}_{Reduced\;\left(\%\right)}}=\frac{\left(M_{{CO2}_{Electric}}-M_{{CO2}_{Hbrid}}\right)}{M_{{CO2}_{Electric}}}$$ (8)

**TABLE 1:** ${EF}_{{CO}_{2}}$ of different fuel combustions to generate 1 kWh of electrical energy (IEA, 2016).

Parameter	Fuel type		
${EF}_{{CO}_{2}}$ (kgCO_2_/kWh)	Coal	Oil	Natural gas
	0.95	0.75	0.55

${\text{EF}}_{{\text{CO}}_{2}}$ : CO_2_ emission factor (kgCO_2_/kWh), IEA: International Energy Agency.

### 2.4 Financial return analysis

The financial return resulting from saving energy and reducing carbon emissions in the drying process was calculated from the following equations:

$${MS}_{year}=R_{usage}\times E_{{S/}_{year}}\times P_{kWh}$$ (9)

where ${MS}_{year}$ is the amount of money saved per year; $R_{usage}$ is the ratio of the working period to the dryer used in a year, given as 0.1–1.0 (Elhage et al., 2018); and $P_{kWh}$ is the price of energy saved (price/kWh) in each country, where the Egyptian tariff of kWh according to the Ministry of Electricity and Energy costs 1.6 Egyptian pounds (EGP), which is equal to USD 0.032.

Furthermore, the money obtained from the earned carbon credit, which is obtained from the quantified CO_2_ mitigation (reduced amount of CO_2_ emitted) of the dryer system, can be calculated from the following equation:

$${CO}_{2Credit}=M_{CO2\;Reduced}\times D$$ (10)

where D is the price value of the carbon credit, which ranges from 20 to 30 USD, with an average of 26 USD/ton of CO_2_ (Nayak et al., 2011; Luxmore et al., 2013; Desa et al., 2020; Carbon Footprint Center, 2024).

### 2.5 Statistical analysis

Data were statistically handled to determine the average ± standard deviation (Avg. ± SD) of triplicates. One-way analysis of variance (ANOVA) was used to conduct statistical analysis at a 0.05 level of significance using SPSS software for Windows (Version 21) (SPSS, IBM Corporation, Armonk, New York, United States).

## 3 Results and discussions

Harnessing various forms of solar energy has become a necessity in light of the severe CC resulting from the GHGs emitted in huge quantities by the industrial and agricultural sectors. Therefore, solar drying has become inevitable considering these severe CCs to reduce the GHGs through traditional non-renewable energy consumption reduction. Hence, this will lead to a reduction in the carbon footprint of the drying process. At the same time, it enhances the quality of dried products at little or no cost compared to traditional industrial drying, which is costly, environmentally harmful, and of low-quality outdoor or shade drying. Table 2 provides an overview of the results of experimental measurements of the drying experiment for thyme, lavender, lemongrass, marjoram, sage, and basil using three different drying systems, TE, HS_TEE_, and HS_PVSE_ dryers at 30, 40, and 50°C, respectively.

**TABLE 2:** Drying temperature, weight, moisture content, drying time, microbial load, and essential oil for thyme, lavender, lemongrass, marjoram, sage, and basil under drying systems.

Sample	Input drying data			Output drying data			Quality parameter	
	Temp. (°C)	W_I_ (kg)	MC_I_ (%)	W_F_ (kg)	MC_F_ (%)	DT (h)	ML (CFU/g)	EO (%)
Thyme	30	40	62.60	14.96	23.40	40	6.23 ± 0.32	1.30 ± 0.17
	40	40	62.30	15.08	23.50	26	6.18 ± 0.17	1.73 ± 0.29
	50	40	62.20	15.12	23.50	18	7.3 ± 0.33	0.41 ± 0.02
Lavender	30	40	70.90	11.64	20.60	54	5.08 ± 0.11	2.47 ± 0.18
	40	40	71.00	11.6	20.60	30	4.17 ± 0.57	2.76 ± 0.24
	50	40	70.90	11.64	20.60	22	5.9 ± 0.49	1.16 ± 0.083
Lemongrass	30	40	69.20	12.32	21.30	40	6.07 ± 0.32	1.55 ± 0.02
	40	40	68.40	12.64	21.60	24	4.74 ± 0.20	1.96 ± 0.04
	50	40	69.20	12.32	21.30	16	5.95 ± 0.10	1.10 ± 0.05
Marjoram	30	40	73.00	10.8	19.70	42	5.85 ± 0.52	2.60 ± 0.12
	40	40	73.00	10.8	19.70	24	5.4 ± 0.20	3.40 ± 0.11
	50	40	72.80	10.88	19.80	18	6.6 ± 0.08	1.58 ± 0.07
Sage	30	40	75.30	9.8	18.50	38	4.8 ± 0.34	2.39 ± 0.06
	40	40	75.50	9.8	18.50	28	4.0 ± 0.22	2.95 ± 0.03
	50	40	75.80	9.68	18.30	20	4.3 ± 0.27	1.91 ± 0.07
Basil	30	40	84.80	6.08	12.90	58	5.11 ± 0.26	1.36 ± 0.11
	40	40	84.90	6.04	12.80	46	4.85 ± 0.49	1.69 ± 0.06
	50	40	84.50	6.20	13.1	32	5.23 ± 0.31	0.86 ± 0.04

Temp: drying temperature (°C); W_I_: initial weight of fresh material (kg/batch); MC_I_: initial moisture content (wb, %); W_F_: final weight of dried material (kg); MC_F_: final moisture content (wb, %); DT: drying time (h); ML: microbial load (CFU/g); EO: essential oil (%).

These measurements include basic data for drying input and output, the energy consumption data for the drying system, and the microbial load (ML) (CFU/g) and essential oil (EO) (%). The loading capacity of the dryers under study was used, as the initial weight was 40 kg/batch for all samples. Furthermore, in the input data, the initial moisture content values (MC_I_, %) were greatly similar when the drying process began for each plant at each drying temperature 30, 40, and 50°C. Once the drying process began till it was completed, for each plant, the behavior of the drying kinetics was uniform at each temperature. This is proven by the results of the drying process outcomes, where the final weight (W_F_, kg) and final moisture content (MC_F_, %) data constantly decreased until the weight stabilized. This is due to the evaporation of free water from the surfaces of the different plants because of their exposure to drying temperatures, where the average values of the final weight (W_F_, kg) as a result of the drying process were 15.05 ± 0.08, 11.63 ± 0.02, 12.43 ± 0.18, 10.83 ± 0.05, 9.76 ± 0.07, and 6.11 ± 0.08 kg for thyme, lavender, lemongrass, marjoram, sage, and basil, respectively. As the same deterioration behavior was observed for weight, the MC_I_ value of all samples decreased till it reached the values of the MC_F_, as listed in Table 2. The drying time (DT) differed at each drying temperature and plant due to the differences in the intensity of drying temperatures (30, 40, and 50°C) and the variation in both the structure and moisture content of each plant. The shortest DT was recorded at 50°C, and the longest DT was observed at 30°C with all the drying systems. Moreover, the quality parameters after the drying process achieved their best values at 40°C for all samples, where the best ML was achieved at 40°C, while the best EO ratio was noted at 40°C with values of 3.40, 2.95, 2.76, 1.96, 1.73, and 1.69% for marjoram, sage, lavender, lemongrass, thyme, and basil, respectively. This is consistent with the results obtained by Amer et al. (2024) and Ibrahim et al. (2023b) for the best drying temperature of 40°C for the quality characteristics of some medicinal and aromatic plants. Regarding energy consumption, the world struggles to meet its energy needs, considering the high cost of traditional energy and environmental warnings toward reducing GHG emissions. Hence, energy consumption is now under the microscope in terms of the source and the amount of energy consumed. Therefore, Table 3 shows the estimated values of the energy consumed/batch, daily, and year to complete the drying process using the TE and HS_TEE_, dryers. The results of the energy consumed (kWh) to complete the drying process pointed to a large difference in the energy consumed between the TE and HS_TEE_ dryers. The TE dryer consumed the highest amount of energy during the drying process and at 50°C, with values of 101.87, 68.76, 66.08, 55.43, and 55.43 kWh/batch for basil, lavender, sage, thyme, and marjoram, respectively.

**TABLE 3:** Energy consumption analysis data/batch, day, and year of the traditional electric dryer (TE dryer) and hybrid solar dryer that uses traditional electric energy (HS_TEE_ dryer) for lavender, lemongrass, marjoram, sage, and basil samples.

Sample	Temp. (°C)	DT (h)	Energy consumption (kWh)					
			TE dryer			HS_TEE_ dryer		
			TE_c_ (kWh)/			TE_c_ (kWh)/		
			Batch	Day	Year	Batch	Day	Year
Thyme	30	40	48.28	9.66	2,317.54	24.67	4.93	1,184.16
	40	26	55.43	17.06	4,093.44	33.04	10.17	2,439.88
	50	18	55.43	24.64	5,912.75	32.78	14.57	3,496.53
Lavender	30	54	65.10	9.65	2,314.81	34.22	5.07	1,216.71
	40	30	62.63	16.70	4,008.58	34.78	9.27	2,225.92
	50	22	68.76	25.00	6,000.61	43.02	15.64	3,754.47
Lemongrass	30	40	45.55	9.11	2,186.50	22.57	4.51	1,083.36
	40	24	51.47	17.16	4,117.36	30.86	10.29	2,468.80
	50	16	48.87	24.43	5,864.04	27.72	13.86	3,326.40
Marjoram	30	42	48.28	9.20	2,207.18	26.93	5.13	1,231.09
	40	24	51.47	17.16	4,117.36	30.86	10.29	2468.80
	50	18	55.43	24.64	5,912.75	32.78	14.57	3,496.53
Sage	30	38	47.52	10.00	2,400.76	22.09	4.65	1,116.13
	40	28	57.34	16.38	3,932.09	33.51	9.57	2,297.83
	50	20	66.08	26.43	6,343.58	36.30	14.52	3,484.80
Basil	30	58	68.25	9.41	2,259.31	34.50	4.76	1,142.07
	40	46	95.90	16.68	4,002.82	56.97	9.91	2,377.88
	50	32	101.87	25.47	6,112.08	59.70	14.93	3,582.00

Tem: drying temperature (°C); DT: drying time (h); TE_c_: total energy consumption (kWh); TE dryer: traditional electric dryer; HS_TEE_ dryer: hybrid solar dryer based on traditional electric energy.

**FIGURE 2:**
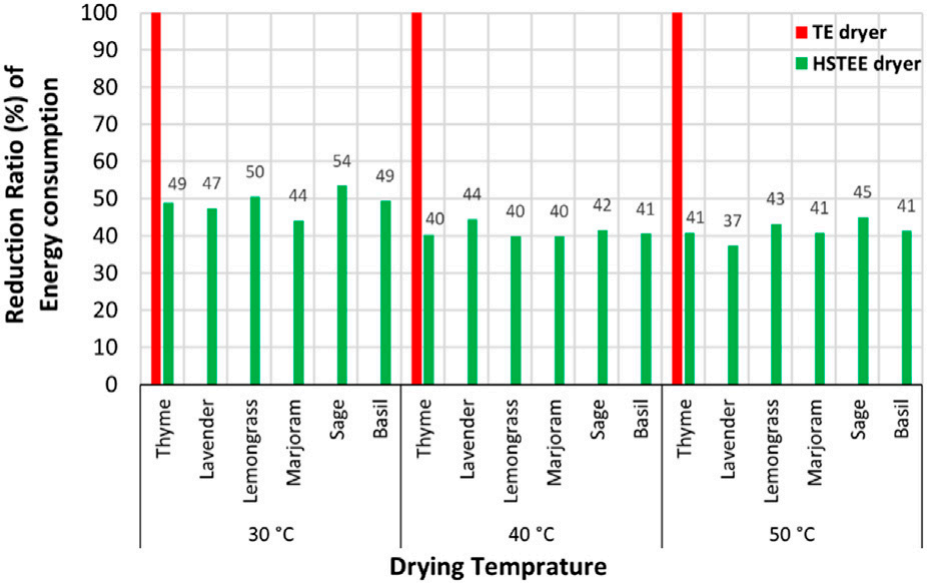
Schematic representation of the energy reduction ratio of the HS_TEE_ dryer compared to the TE dryer.

In contrast, the HS_TEE_ dryer consumed a high amount of traditional electrical energy at 50°C with values of 59.70, 43.02, 36.30, 32.78, and 32.78 kWh/batch for basil, lavender, sage, thyme, and marjoram, respectively. The HS_PVSE_ dryer consumed the same energy as the HS_TEE_ dryer at all drying temperatures used, but the source of electrical energy was generated from the renewable PV panel system without CO_2_ emissions. On the contrary, the lowest values of energy consumed by TE, HS_TEE_, and HS_PVSE_ dryers were recorded at 30°C. The results of the energy consumed for both TE and HS_TEE_ dryers highlighted the significant difference in the energy consumed, whether over the daily or the annual energy consumption at operating the dryer continuously. The TE dryer consumed the highest amount of energy per day (8 working hours) and at 50°C with values 26.43, 25.47, 25, 24.64, 24.64, and 24.43 kWh/day for sage, basil, lavender, thyme, marjoram, and lemongrass, respectively. In contrast, the highest energy consumed per day was noted at 50°C for the HS_TEE_ dryer but with lower values than those obtained by the TE dryer, with values of 15.64, 14.93, 14.57, 14.52, 14.52, and 13.86 kWh/day for lavender, basil, thyme, marjoram, sage, and lemongrass, respectively. Hence, the results of the energy consumption/day were strong indicators for calculating the energy consumption/year for all dryers. The highest rate of energy consumption was achieved annually at 50°C for the TE dryer, with a value of 6,343.58 kWh/year for the sage sample. In contrast, the sage sample at the HS_TEE_ dryer and 50°C consumed energy with a value of 3,484.80 kWh/year. The lowest energy consumption/year was noted at the HS_TEE_ dryer and 30°C for the lemongrass sample with a value of 1,083.36 kWh/year, while the lowest energy consumption/year for the TE dryer was 2,186.50 kWh/year at 30°C for the lemongrass sample. This large difference in the energy consumed between TE and both HS_TEE_ and HS_PVSE_ dryers may be due to the ability of the hybrid solar system and photovoltaic panels to convert solar energy into thermal and electrical energy, which is useful for drying operations. This leads to the efficient use of energy, the best drying time, and high quality. The energy reduction ratio of the HS_TEE_ dryer compared to the TE dryer is shown in Figure 2. The highest ratio of CO2 mitigated was noted for lavender, thyme, basil, lemongrass, and sage samples with values ranging from 45% to 54% at 30, and 50°C. The lowest ratio of energy reduction for the HS_TEE_ dryer ranged between 37% and 40% for lavender, marjoram, lemongrass, and thyme at 40°C and 50°C. Finally, the consumption of traditional energy to operate dryers can be zero when relying on operating the HS_PVSE_ dryer that is based on the photovoltaic panels as a source of electrical energy.

**FIGURE 3:**
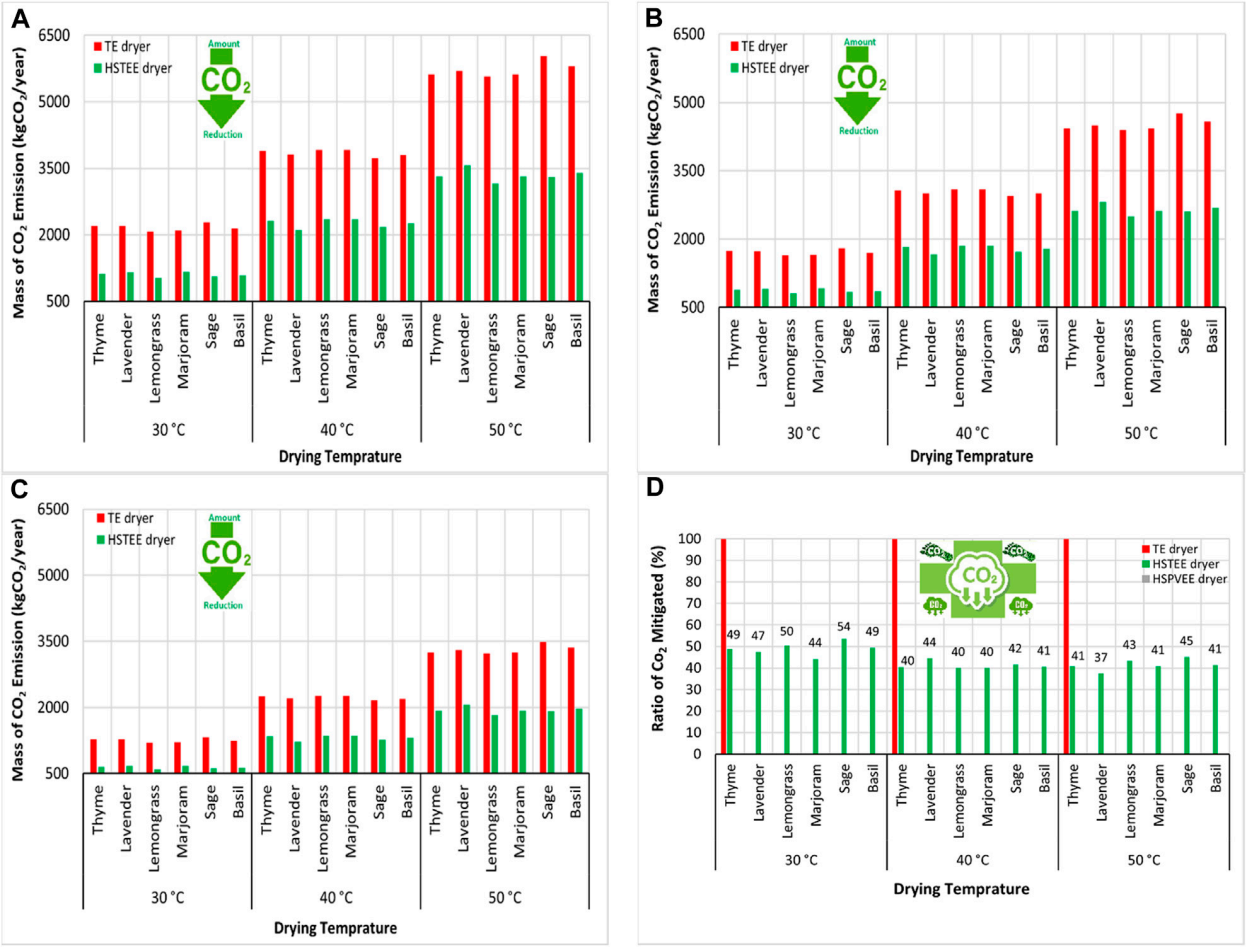
Schematic representation of the CO_2_ mitigated/year using the HS_TEE_ dryer and hybrid solar dryer based on proposed photovoltaic solar energy (HS_PVSE_ dryer) vs. TE dryer at different CO_2_ emission factors. **(A)** Coal emission factor (kgCO_2_/kWh), **(B)** oil emission factor (kgCO_2_/kWh), **(C)** natural gas emission factor (kgCO_2_/kWh), and **(D)** annual CO_2_ mitigated ratio.

The amount of CO_2_ production, which contributes to GHG emissions, is considered one of the most important indicators in calculating the energy consumed or embodied to carry out the drying process. Hence, a relationship has been linked between the conventional electrical energy consumed generated from different fuel sources such as coal, oil, and natural gas and the CO_2_ emission factor for each type of fuel source. Then, the amounts of CO_2_ emitted for the drying process using TE and HS_TEE_ dryers were calculated and estimated. As a result, Figure 3 shows the amount and the ratio of CO_2_ produced and mitigated/year using the TE and HS_TEE_ dryers at different CO_2_ emission factors for coal, oil, and natural gas. The results proved that in the case of using coal to generate traditional electricity, the amounts of CO_2_ produced annually because of the use of TE dryers recorded the highest values of the amounts of CO_2_ at 50, 40, and 30°C, where the mass of CO_2_ produced using the TE dryers ranged from 2,146.34 to 6,026.40 kgCO_2_/kWh/year. The highest mass of CO_2_ produced was 6,026.40 kgCO_2_/kWh/year for the sage sample at 50°C, followed by basil, lavender, thyme, marjoram, and lemongrass dried samples at 50°C, which recorded the highest amounts of CO_2_ produced with values of 5,806.48, 5,700.58, 5,617.11, 5,617.11, and 5,570.84 kgCO_2_/kWh/year, respectively.

In the same way, oil and natural gas used in generating traditional electricity came in 2^nd^ and 3^rd^ place, respectively, in terms of the amounts of CO_2_ emissions produced as a result of the drying process using the TE dryer. Their highest values were recorded at 50°C, i.e., 4,757.69 and 3,488.97 kgCO_2_/kWh/year for sage samples. Moreover, the lowest values for CO_2_ emission production were found at 30°C for all types of fuels.

On the other hand, the HS_TEE_ dryer achieved low emissions of CO_2_ from all types of fuel (coal, oil, and natural gas) compared to the TE dryer due to the efficient use of solar energy. Here, the lowest amounts of CO_2_ produced were 1,029.19, 812.52, and 595.85 kgCO_2_/kWh/year using coal, oil, and natural gas, respectively, at 30°C for lemongrass. The highest amounts of CO_2_ produced were noted at 50°C for lavender samples, i.e., 3,566.75, 2,815.85, and 2,064.96 kgCO_2_/kWh/year using coal, oil, and natural gas, respectively. This is compared to the same values for the amounts of CO_2_ emitted at the same temperature and the same type of samples using the TE dryer, which were 5,700.58, 4,500.46, and 3,300.34 kgCO_2_/kWh/year using coal, oil, and natural gas, respectively. This large difference in the amounts of CO_2_ emitted between TE and HS_TEE_ dryers leads to achieving large percentages of mitigating the CO_2_ emitted, as shown in Figure 3. The highest ratio of CO_2_ mitigated was noted for lavender, thyme, basil, lemongrass, and sage samples with values ranging from 45% to 54% at 30, and 50°C. This is compared to the original ratio of CO_2_ emitted (100%) using the TE dryer. The other ratio of CO_2_ mitigated using the HS_TEE_ dryer ranged between 37% and 44% for all samples at 30, 40, and 50°C. Finally, the mitigated ratio of CO_2_ emissions can be zero, when relying on operating the HS_PVSE_ dryer that is based on photovoltaic panels as a source of electrical energy.

### 3.1 Save energy and money

**FIGURE 4:**
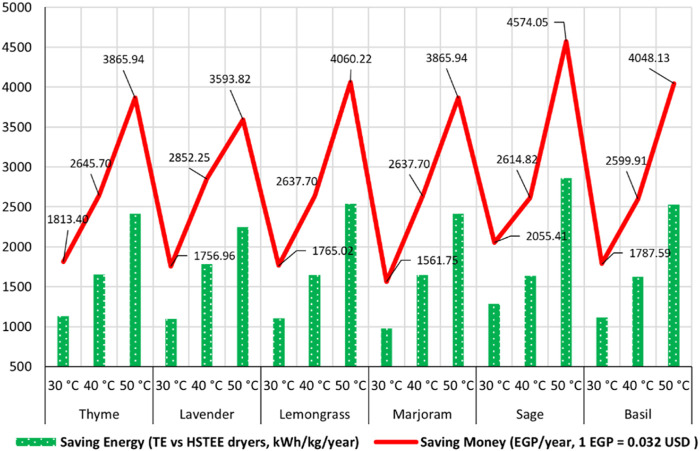
Energy saving by the HS_TEE_ dryer and saving cost.

One of the most important advantages of using solar energy and harnessing it by using solar and hybrid solar dryers lies in their ability to save energy, which, in turn, leads to saving money. The traditional electrical energy consumed by the TE dryer pays the cost of its consumption bill with more money. Therefore, naturally, the energy saved using hybrid solar dryers turned into saving money, as shown in Figure 4. All the data obtained in Figure 4 indicate that the dryers operate at full capacity for 8 h a day, 20 working days a month, and 12 months throughout the year. The highest energy-saving values, corresponding to the highest cost-saving values, were achieved at 50°C for sage, lemongrass, basil, thyme, marjoram, and lavender with values of 4,574.05, 4,060.22, 4,048.13, 3,865.94, 3,865.94, and 3,593.82 EGP/year, respectively. The drying temperature of 40°C also achieved average saving values for both energy and money saved. In addition to the best quality parameters achieved at 40°C, the amounts of energy saved were translated into money saved, with values of 2,852.25, 2,645.7, 2,637.7, 2,637.7, 2,614.82, and 2,599.91 EGP/year at 40°C for lavender, thyme, lemongrass, marjoram, sage, and basil, respectively, while the lowest saving amounts of energy and money were achieved at 30°C.

### 3.2 CO_2_ mitigation and saving money

**FIGURE 5:**
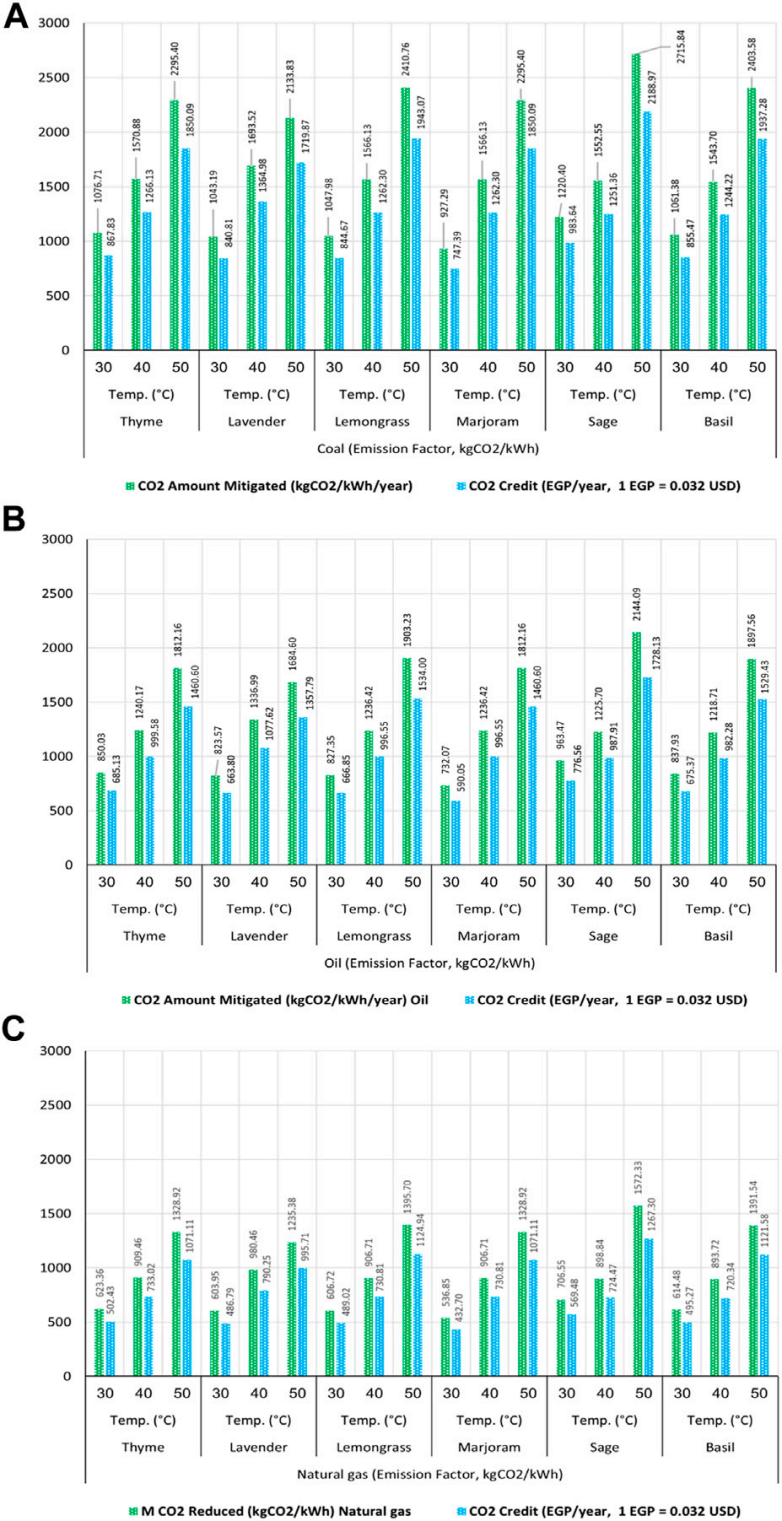
Amount of CO_2_ mitigated (kgCO_2_/year) vs. saving money (EGP): **(A)** coal emission factor (kgCO_2_/kWh), **(B)** oil emission factor (kgCO_2_/kWh), and **(C)** natural gas emission factor (kgCO_2_/kWh).

The results obtained showed that the percentage of CO_2_ reduction for the drying systems used practically depends on the type of dryer and fuel used to produce electricity. The results showed a reduction in the percentage of CO_2_ emissions by a rate ranging from 37% to 54% when using the HS_TEE_ dryer. In the case of using the HS_PVSE_ dryer, the ratio of reducing CO_2_ emissions can reach 100% due to the operation of photovoltaic panels to generate electricity. Therefore, the carbon credit model approved for monetary incentives allowed transactions between agencies and individuals to participate in reducing the carbon footprint and, at the same time, finance reduction plans globally. To confirm this, the environmental impact and financial return were analyzed in terms of monitoring and estimating the amounts of CO_2_ mitigated using the HS_TEE_ vs. TE dryers. Furthermore, the amount of money saved as a result of CO_2_ mitigation was calculated. The highest amounts of mitigated CO_2_ were achieved by using coal as a source of electricity generation, as shown in Figure 5. As the mitigated amounts of CO_2_ ranged from 927.29 to 2,715.84 kgCO_2_/kWh/year at 30, 40, and 50°C, the highest mitigated amounts of CO_2_ were observed at 50°C, with values of 2,133.83, 2,295.40, 2,295.40, 2,403.58, 2,410.76, and 2,715.84 kgCO_2_/kWh/year in exchange for saving money, with values of 1,719.87, 1,850.09, 1,850.09, 1,937.29, 1,943.07, and 2,188.97 EGP/year for lavender, thyme, marjoram, basil, lemongrass, and sage, respectively. The average values of CO_2_ credit were achieved at the best drying temperature of 40°C, where the amounts of mitigated CO_2_ were estimated at values of 1,543.70, 1,552.55, 1,566.13, 1,566.13, 1,570.88, and 1,693.52 kgCO_2_/kWh/year, and money savings were achieved at values of 1,244.22, 1,251.36, 1,262.30, 1,262.30, 1,266.13, and 1,364.98 EGP/year for basil, sage, lemongrass, marjoram, thyme, and lavender, respectively. Finally, the lowest amounts of CO_2_ mitigation were achieved at 30°C, as well as the lowest savings in money, as its value ranged from 747.39 to 983.64 EGP/year. The same approach was used when using oil as a source of electricity generation but with lower CO_2_ mitigation and money-saving values because of the emission factor of oil being lower than that of coal. The highest values of the amounts of mitigated CO_2_ and the corresponding savings in money were recorded at 50°C. This is because of the highest decrease in the energy consumed at 50°C. At 40°C, the values of the amounts of CO_2_ mitigated and the corresponding money savings ranged from 1,218.71 to 1,336.99 kgCO_2_/kWh/year and 982.28 to 1,077.92 EGP/year, respectively, as shown in Figure 5.

**FIGURE 6:**
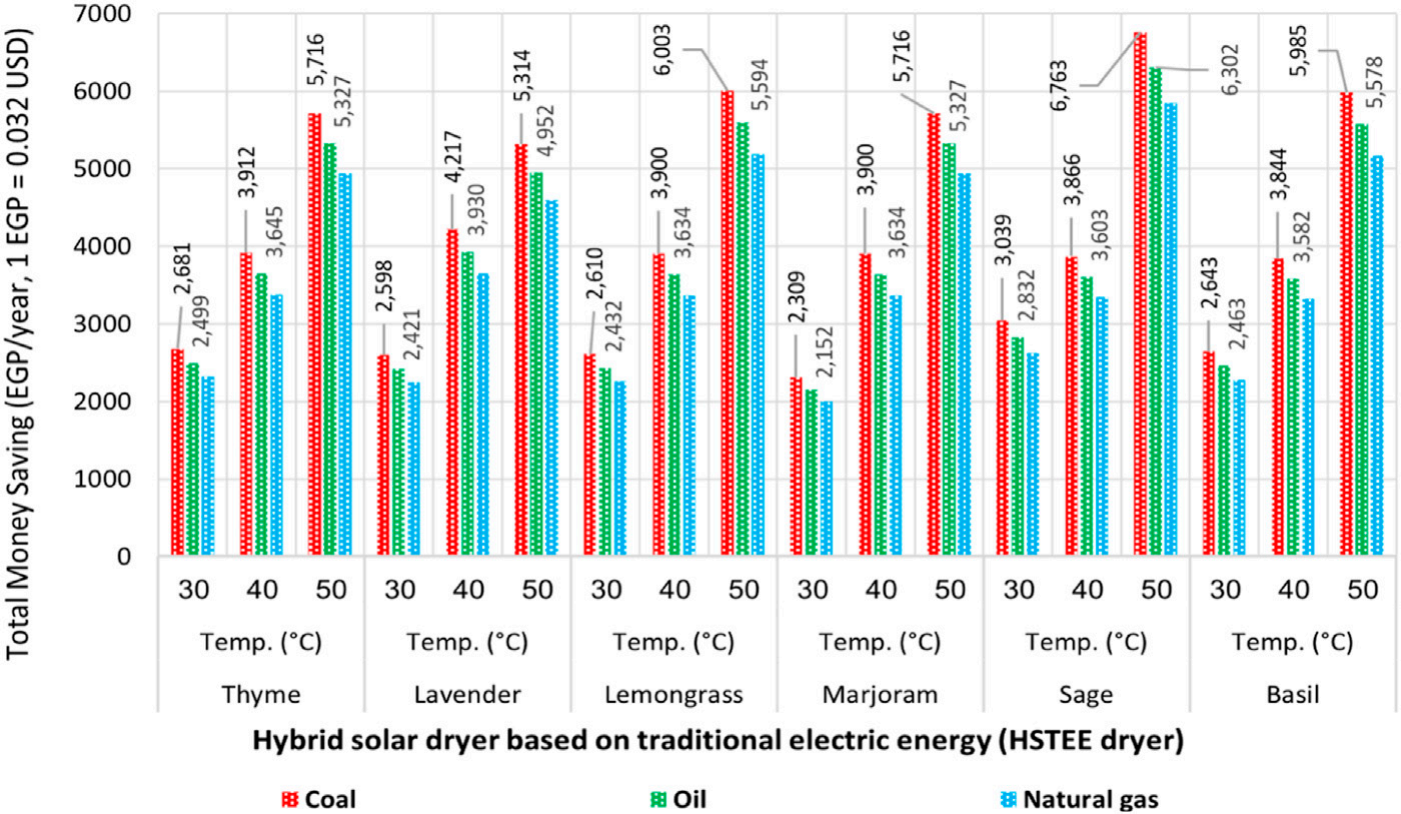
Schematic representation of the total money saving from energy reduction and CO_2_ credit by the hybrid solar dryer uses traditional electric energy (HS_TEE_ dryer).

Natural gas has also been characterized by the same behavior of mitigating amounts of CO_2_ and saving money for both coal and oil but in smaller quantities due to the emission factor of natural gas being as low as possible. The highest amounts of CO_2_ mitigation and money saving were achieved at 50, 40, and 30°C, as shown at the bottom of Figure 5. The final financial analysis, which combines saving money by reducing energy consumption and saving money resulting from mitigating the amount of CO_2_ emitted (carbon credit or carbon footprint certification) using the HS_TEE_ dryer, is shown in Figure 6. Coal, as a fuel source of generating electrical energy, achieved the highest financial return resulting from energy savings + carbon credit due to the high CO_2_ emission factor of coal. The highest financial return is achieved at 50°C with values of 5,313.69, 5,716.04, 5,716.04, 5,985.41, 6,003.29, and 6,763.03 EGP/year for lavender, thyme, marjoram, basil, lemongrass, and sage, respectively. These values decreased at the lower drying temperatures of 30°C and 40°C. This is due to the decrease in energy consumption and, consequently, the decrease in the amounts of mitigated CO_2_ compared to the higher drying temperature of 50°C.

**FIGURE 7:**
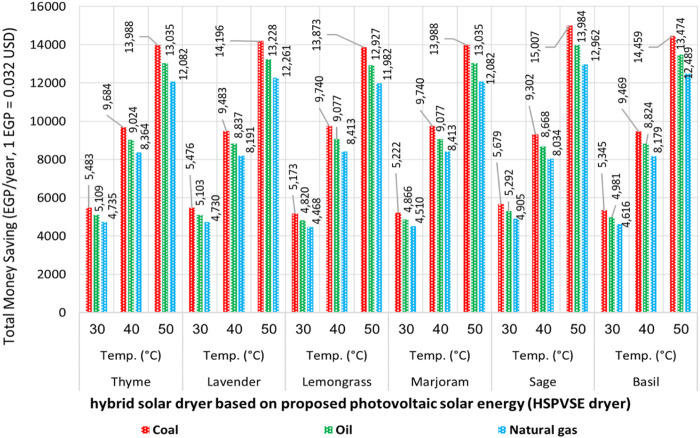
Schematic representation of the total money saved from energy reduction and CO_2_ credit using the hybrid solar dryer based on the proposed HS_PVSE_ dryer.

Then, oil as a fuel source for generating traditional electrical energy came in the second place in terms of providing the highest financial return as it ranged from 4,951.61 to 6,302.19, 3,602.73 to 3,929.87, and 2,151.79 to 2,831.97 EGP/year at 50, 40, and 30°C, respectively. In the same way, natural gas achieved the third place in terms of providing the highest financial return because it has the lowest CO_2_ emission factor of 0.55 kgCO_2_/kWh, according to EA (2016 **)**. To achieve zero CO_2_ emission and provide a greater financial return than the HS_TEE_ dryer, the third option was to harness solar energy by converting it into electrical and thermal energy. Figure 7 shows the effect of using the HS_PVSE_ on the value of saving money, resulting from reducing traditional energy consumption and zero CO_2_ emission. It achieved a 100% reduction in energy consumption and then reduced CO_2_ emissions by 100%, which led to a 100% financial return from both energy reduction and carbon credit. The highest financial return was achieved in the CO_2_ emission factor for coal, oil, and natural gas at 30, 40, and 50°C, respectively.

The highest financial returns were observed at 50°C, with values ranging from 13,872.56 to 15,007.02, 12,927.28 to 13,984.43, and 11,981.99 to 12,961.85 EGP/year for coal, oil, and natural gas, respectively. In contrast, the results of the highest financial return of using the HS_TEE_ dryer were with values ranging from 5,313.69 to 6,763.03, 4,951.61 to 6,302.19, and 4,589.53 to 5,841.35 EGP/year at 50°C for coal, oil, and natural gas, respectively. The drying temperature of 40°C came in the second place in terms of saving money as it achieved values ranging from 9,302.15 to 9,740.44, 8,668.30 to 9,076.72, and 4,467.67 to 4,905.47 EGP/year for coal, oil, and natural gas emission factors, respectively. In contrast, the overall values of saving money using the HS_TEE_ dryer ranged from 3,844.13 to 4,217.23, 3,602.73 to 3,929.87, and 2,309.14 to 3,039.05 EGP/year at 40°C for coal, oil, and natural gas emission factors, respectively. Finally, the HS_PVSE_ dryer at 30°C achieved the lowest financial returns, with values ranging from 5,172.59 to 5,679.47, 4,820.13 to 5,292.47, and 4,467.67 to 4,905.47 EGP/year, compared to values obtained for the HS_TEE_ dryer, which range from 2,309.14 to 3,039.05, 2,151.79 to 2,831.97, and 1,994.45 to 2,624.89 EGP/year for coal, oil, and natural gas emission factors, respectively.

## 4 Conclusion

Solar energy is one of the most important renewable energies used for reducing the effects of severe CC. Based on this, drying using solar energy, whether using solar or hybrid solar dryers, is one of the most promising applications of solar thermal technology for replacing traditional thermal technology that contributes to increasing the phenomenon of severe CC. Therefore, analyzing energy consumption is crucial for selecting the right dryer for achieving drying goals and financial returns. In this study, the HS_TEE_ and HS_PVSE_ dryers effectively contributed to preserving the environment because they reduced the demand for energy consumption, mitigated CO_2_ emissions, and achieved a financial return. The best quality of dried medicinal and aromatic plant samples in terms of maintaining the highest percentage of EO and reducing the ML was observed at 40°C using both HS_TEE_ and HS_PVSE_ dryers. However, for lavender, marjoram, lemongrass, and thyme at 40°C and 50°C, the lowest energy reduction ratio obtained using the HS_TEE_ dryer varied from 37% to 40%. When using photovoltaic panels as an electrical energy source to operate the HS_PVSE_ dryer, dryers can be operated with 0% traditional energy consumption. In addition, the amounts of CO_2_ mitigated and the associated cost reductions at 40°C ranged from 1,218.71 to 1,336.99 kgCO_2_/kWh/year and 982.28 to 1,077.92 EGP/year, respectively. For coal, oil, and natural gas emission factors at 40°C, the overall saving values obtained using the HS_TEE_ dryer ranged from 3,844.13 to 4,217.23, 3,602.73 to 3,929.87, and 2,309.14 to 3,039.05 EGP/year, respectively. From these findings, it can be concluded that the HS_TEE_ and HS_PVSE_ dryers could contribute to environmental conservation by saving energy and reducing GHG emissions and also provide a financial return, thus achieving sustainable development goals.

## Data Availability

The original contributions presented in the study are included in the article/Supplementary Material; further inquiries can be directed to the corresponding author.
