# Corrigendum: Applied insight: studying reducing the carbon footprint of the drying process and its environmental impact and financial return

**DOI:** 10.3389/fbioe.2024.1431416

**Published:** 2024-06-12

**Authors:** Ayman Ibrahim, Alia Amer, Islam Elsebaee, Amr Sabahe, Mariam A. Amer

**Affiliations:** ^1^ Bioengineering Department, Agricultural Engineering Research Institute (AEnRI), Agricultural Research Center (ARC), Giza, Egypt; ^2^ Medicinal and Aromatic Plants Research Department, Horticulture Research Institute, Agricultural Research Center (ARC), Giza, Egypt

**Keywords:** drying, solar energy, hybrid solar dryer, energy consumption, greenhouse gas emissions, carbon footprint

In the published article, there was an error in Figures 2, 3D as published. There was an error in the arrangement of ratio values on the columns. The corrected Figures 2, 3D appear below.

**FIGURE 2:**
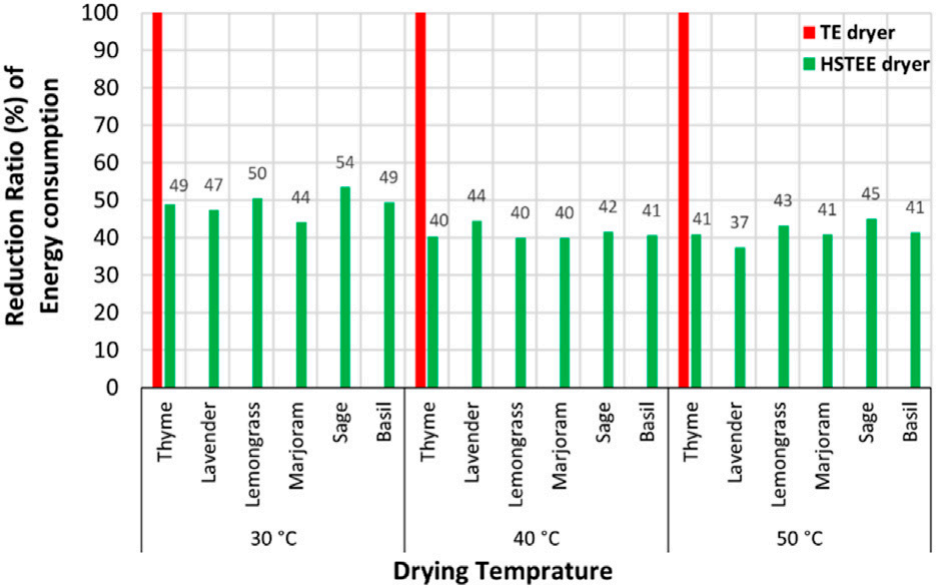
Schematic representation of the energy reduction ratio of the HSTEE dryer compared to the TE dryer.

**FIGURE 3:**
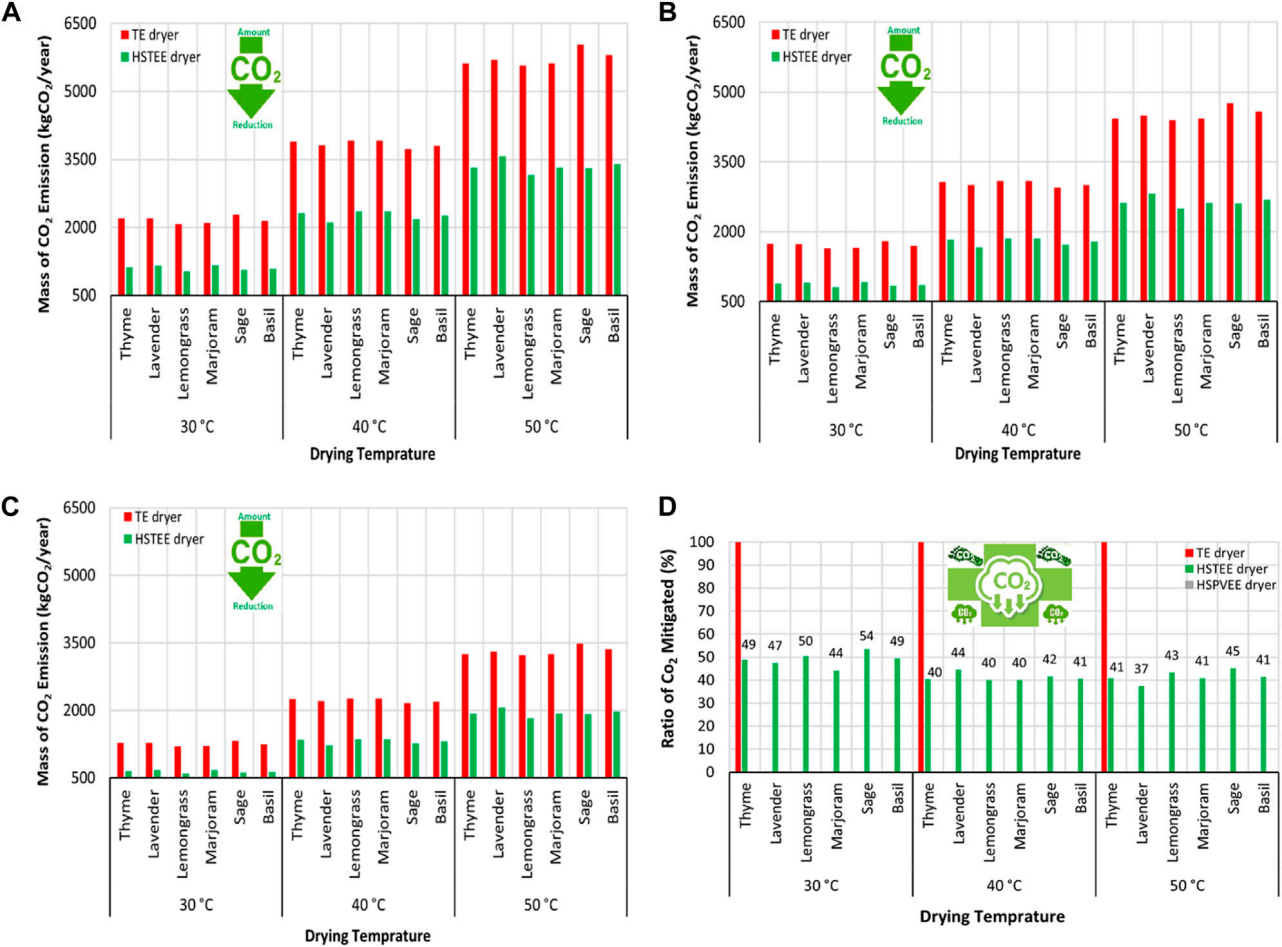
Schematic representation of the CO_2_ mitigated/year using the HSTEE dryer and hybrid solar dryer based on proposed photovoltaic solar energy (HSPVSE dryer) vs. TE dryer at different CO_2_ emission factors. **(A)** Coal emission factor (kgCO_2_/kWh), **(B)** oil emission factor (kgCO_2_/kWh), **(C)** natural gas emission factor (kgCO_2_/kWh), and **(D)** annual CO_2_ mitigated ratio.

In the published article, there was an error. As a result of modifying Figures 2, 3D, it was necessary to amend paragraphs in the Abstract, Results and discussions, and Conclusion sections.

A correction has been made to **Abstract.** This sentence previously stated:

“The highest CO_2_ mitigated ratio using the HS_TEE_ dryer was recorded in thyme, marjoram, and lemongrass samples, with values ranging from 45% to 54% at 30, 40, and 50°C.”

The corrected sentence appears below:

“The highest CO_2_ mitigated ratio using the HS_TEE_ dryer was recorded in lavender, thyme, basil, lemongrass, and sage samples with values ranging from 45% to 54% at 30, and 50°C.”

A correction has been made to **3 Results and discussions**, Paragraph 3. This sentence previously stated:

“The highest ratio of energy reduction for the HS_TEE_ dryer compared to the TE dryer was recorded for thyme samples at 30, 40, and 50°C with values of 49%, 50%, and 54%, respectively. The lowest ratio of energy reduction for the HS_TEE_ dryer ranged between 37% and 40% for basil, lavender, and sage at 30°C and 40°C.”

The corrected sentence appears below:

“The highest ratio of CO_2_ mitigated was noted for lavender, thyme, basil, lemongrass, and sage samples with values ranging from 45% to 54% at 30, and 50°C. The lowest ratio of energy reduction for the HS_TEE_ dryer ranged between 37% and 40% for lavender, marjoram, lemongrass, and thyme at 40 °C and 50°C.”

A correction has been made to **Results and discussions**, Paragraph 6. This sentence previously stated:

“The highest ratio of CO_2_ mitigated was noted for thyme, marjoram, and lemongrass samples with values ranging from 45% to 54% at 30, 40, and 50°C.”

The corrected sentence appears below:

“The highest ratio of CO_2_ mitigated was noted for lavender, thyme, basil, lemongrass, and sage samples with values ranging from 45% to 54% at 30, and 50°C.”

A correction has been made to **4 Conclusion**, Paragraph 1. This sentence previously stated:

“However, for sage, lavender, and basil at 30°C and 40°C, the lowest energy reduction ratio obtained using the HS_TEE_ dryer varied from 37% to 40%.”

The corrected sentence appears below:

“However, for lavender, marjoram, lemongrass, and thyme at 40°C and 50°C, the lowest energy reduction ratio obtained using the HS_TEE_ dryer varied from 37% to 40%.”

The authors apologize for these errors and state that they do not change the scientific conclusions of the article in any way. The original article has been updated.

